# ‘I Got Dentures in, So at Least I Can Eat Now’: Aboriginal and Torres Strait Islander Peoples' Perceptions of and Experiences With Removable Dentures

**DOI:** 10.1002/hpja.70259

**Published:** 2026-10-20

**Authors:** Ria Aiyar, Gina L. Guzzo, Sonia Nath, Nicolas Reid, Kostas Kapellas, Johanna Groundwater, Lisa M. Jamieson

**Affiliations:** ^1^ Australian Research Centre for Population Oral Health, School of Dentistry, Adelaide University Adelaide South Australia Australia; ^2^ The Royal Flying Doctor Service (SA/NT) Adelaide South Australia Australia

**Keywords:** Aboriginal Australians, health equity, health promotion, mouth rehabilitation, oral health, qualitative research, Torres Strait Islanders

## Abstract

**Introduction:**

Provision of dentures to people with partial or complete tooth loss aims to restore oral health and improve functions such as chewing and speech, and facial appearance. Lived experience perspectives regarding tooth loss and subsequent oral rehabilitation with dentures are underexplored, particularly among Aboriginal and Torres Strait Islander Peoples in Australia, who experience persistent inequities in oral health and access to dental care. The aim of the current study is to understand Aboriginal and Torres Strait Islander adults' perceptions of and experiences with removable dentures.

**Methods:**

The qualitative results are part of a quasi‐experimental study exploring culturally safe dental care among Aboriginal and Torres Strait Islander Peoples (retrospectively registered with the Australian New Zealand Clinical Trials Registry, trial registration: ACTRN12626000046303). Semi‐structured interviews were conducted with 136 Aboriginal and Torres Strait Islander adults and analysed using reflexive thematic analysis.

**Results:**

Three overarching themes were developed: (1) Decision‐making for dentures: Pain, prior treatment and appearance; (2) Unmet needs and expectations of dentures; and (3) Dentures influence social and emotional wellbeing. Together, these themes comprise seven subthemes.

**Conclusion:**

Participants described various impacts including functional challenges related to both tooth loss and denture wearing, limited patient autonomy around denture‐related decision‐making, high costs and improvements to confidence and social and emotional wellbeing after provision of dentures.

**So What?:**

Aboriginal and Torres Strait Islander adults highlight the need for equitable approaches to accessible, affordable and patient‐centred prosthetic care that ensures a holistic focus on quality of life to promote oral health and wellbeing.

## 1 Background

Tooth loss is considered a marker of damage from oral health concerns such as dental caries (tooth decay), periodontal (gum) disease and dental trauma as well as broader systemic and access issues including high costs to restore damaged teeth and long wait times [1]. Previous research provides evidence of the potential link between poor oral health and systemic health, including chronic conditions such as cardiovascular disease, diabetes and mental health [2, 3], with concerns such as Type‐2 diabetes associated with an increased risk of tooth loss [4]. Studies across cultures and contexts have identified various physical and psychosocial impacts after partial or total tooth loss (edentulism) [5, 6, 7] including lowered confidence and satisfaction with appearance intertwined with functional impacts such as challenges related to chewing food or speaking [7].

The impacts of tooth loss may be alleviated through oral rehabilitation—personalised oral therapy aimed at restoring function, health and aesthetics of the mouth [8], including through the use of custom‐made dental prosthetics (e.g., removable dentures; hereafter, dentures). In restoring aesthetics, dentures are one of the most common treatment options in prosthetic dentistry for people with extensive tooth loss, and their implications extend beyond any functional aspects they aim to improve [5]. For example, participants in prior international qualitative studies have described improved function in terms of eating, speaking and better self‐confidence after receiving dentures [6, 9, 10]. While existing research regarding perceptions and experiences of dentures are well‐established, for populations who experience ongoing barriers to accessing oral healthcare such as Aboriginal and Torres Strait Islander Australians, experiences around tooth loss and denture‐wearing are less well‐understood.

### 1.1 Oral Health for Aboriginal and Torres Strait Islander Peoples

In Australia, around 1 in 10 people over the age of 15 have inadequate dentition (less than 21 teeth) with 4% of people aged 15 years or older considered edentulous (lacking any natural teeth or being completely toothless) [11]. Relative to non‐Indigenous Australians, the Aboriginal and Torres Strait Islander population have higher rates of dental disease, including 2.77 times the prevalence of untreated dental caries [12] and are overrepresented in regard to complete tooth loss, particularly among Aboriginal and Torres Strait Islanders aged 55–74 (29.3% vs. 7.7%) [13, 14]. These issues are generally influenced by social determinants of health including the impacts of colonisation [15]. Aboriginal and Torres Strait Islanders largely hold a holistic view of oral health, such that aesthetic‐related or functional impacts of oral health (including tooth loss) may greatly impact their social and emotional wellbeing including feelings of confidence, shame and social participation [16, 17]. Additionally, many Aboriginal and Torres Strait Islanders report several barriers in accessing oral healthcare including discrimination, a lack of culturally safe, trauma‐informed care, high costs due to a lack of subsidised dental services [18] and past experiences of painful or invasive procedures [19].

In relation to tooth loss, access to removable dentures in Australia can be costly, with specific eligibility criteria to be met in order to access subsidised care for dentures, relines and repairs (see SA Dental [20]) highlighting further barriers around access to oral rehabilitation. Of the limited research in this area, one prior study initiated by the Poche Centre for Indigenous Health reported that dentures remain an important treatment option for Aboriginal and Torres Strait Islander Peoples with extensive tooth loss [21]. In this study, a mobile denture service run by an Aboriginal dental technician, prosthodontists and a local oral health team (dentists, oral health therapists, dental assistants) provided Aboriginal participants in rural New South Wales with dentures within a timeframe of 4 days to address the impacts of tooth loss and poorer oral health in communities [21]. While participants in this study reported significant improvements in quality of life, wellbeing and oral health, research in this area remains scarce, providing limited insight into how Aboriginal and Torres Strait Islander adults perceive and experience dentures, including their experiences regarding comfort, function, appearance and access to ongoing denture care. These lived experiences are essential to understand, given the inequities experienced by some Aboriginal and Torres Strait Islander communities in terms of oral health, and the reported barriers to accessing dental care. Understanding these experiences are key in identifying opportunities to improve health equity and community health outcomes. As such, the aim of the current study is to understand Aboriginal and Torres Strait Islander Australian adults' perceptions of and experiences with removable dentures.

## 2 Methods

### 2.1 Design

This qualitative paper reports results from a mixed‐methods, non‐randomised implementation and service‐delivery project which aimed to evaluate the provision of culturally safe dental care for Aboriginal and Torres Strait Islander South Australians. The quasi‐experimental study is registered with the Australian New Zealand Clinical Trials Registry (registration number: ACTRN12626000046303). A protocol detailing the study rationale and methods has been previously published [22]. The broader study involved a pre–post assessment of oral health embedded in routine dental care whereby Aboriginal and Torres Strait Islander participants consented to oral epidemiological examinations and assessment of systemic health biomarkers at baseline (July 2022–December 2023) and 12‐month follow‐up (March 2024–March 2025), with formal dental care received between baseline and follow‐up. Participants involved in the 12‐month follow‐up phase were offered the opportunity to share their experiences with the intervention, dental care and with oral health more broadly through a semi‐structured interview.

### 2.2 Participants and Recruitment

Eligibility criteria for adult participants were: (a) aged 18 years and above; (b) identified as Aboriginal and/or Torres Strait Islander; (c) resided in South Australia at the time of the study. Participants were recruited through convenience and snowball sampling whereby study information was shared with local Aboriginal Community Controlled Health Organisations and via social media. Those interested could then contact the research team and refer eligible friends or family to join the study. Participants were provided with a shopping voucher for their time and contributions to the research.

### 2.3 Ethical Considerations

The study was granted ethical approval by the Aboriginal Health Research Ethics Committee and the University of Adelaide Human Research Ethics Committee (joint approval number: 04‐22‐990). An Indigenous Oral Health Unit Reference Group with Indigenous leadership provided study governance in prioritising cultural authority and cultural safety across the project. The study was informed by the CONSIDER statement for Indigenous health reporting guidelines [23] (Supporting Information). Consistent with the analytic approach (reflexive thematic analysis [24]) member checking was not undertaken, and results were returned to participants and communities through newsletters and community presentations as part of the broader engagement and dissemination process.

### 2.4 Data Analysis

While interviews broadly explored Aboriginal and Torres Strait Islanders' oral health experiences and experiences with the culturally safe dental care study, the present analysis specifically examines the meanings attributed to dentures, regardless of participants' denture status. At the time of interviewing, this included the experiences of those who had removable dentures; people with adjacent experiences who provided reflections as relatives of their family member/s with dentures; and non‐denture wearing participants who spoke to anticipated denture‐related experiences.

For the qualitative component of this research project and associated publications using qualitative data [16, 18], the primary analytic approach employed was reflexive thematic analysis [24]. While another published qualitative study from this project employed template thematic analysis for a structured analysis of predefined topics of interest within the large data corpus (see Nath et al. [25]), the themes in this paper were generated through iterative and interpretative engagement with the dataset as per Braun and Clarke's six phases of reflexive thematic analysis [24]. The first phase involved familiarisation with the data, whereby the first author transcribed all interviews, listened to audio‐recordings and read transcripts noting initial thoughts. In the second phase, codes were generated at the semantic level. During the familiarisation and coding phases, authors noted a coherent and rich pattern of shared meaning around denture experiences. As such, instances where participants oriented to oral prosthesis—including for those who had dentures at the time of the study and those who reflected on dentures despite not being partially dentate or edentulous—were coded. Codes were collated in the third phase followed by theme generation in the fourth phase where initial candidate themes were developed and reviewed. The fifth phase involved defining and finalising themes, and the final phase involved weaving the analytical narrative together.

### 2.5 Reflexivity

In prioritising reflexivity throughout the study, the research team engaged in ongoing discussions led by a Senior Aboriginal Research Officer, who also guided interviewing authors on culturally safe research practices. Guidance was provided around engaging in relational yarning with participants, involving respectful connection and sharing of knowledge and experience through narrative [26]. The authorship team has over 20 years of combined experience in conducting research in the Aboriginal and Torres Strait Islander oral health field including as a non‐Indigenous qualitative researcher (R.A.), non‐Indigenous microbiome scientist (G.L.G.), clinical experience as Aboriginal (N.R.) and non‐Indigenous dentists (S.N., L.J.) and non‐Indigenous oral health therapists (K.K., J.G.). While the clinicians within the authorship team do not have a specialised sub‐focus in prosthodontics, all clinicians are experienced in supporting Aboriginal and Torres Strait Islander patients with tooth loss, extractions and examining and supporting patients with prosthesis care in line with evidence‐based guidelines and culturally safe dental care.

## 3 Results

The overall qualitative data corpus included interviews from 136 Aboriginal and Torres Strait Islander adult participants, with a median age of 46 years (IQR: 32.0–55.3; see Table 1). Most participants were Aboriginal (91.2%), female (58.8%), resided in a metropolitan or inner regional area of South Australia at the time of interview (86%) and were interviewed in‐person (94.9%). About three‐quarters of participants had some degree of tooth loss (76.7%). Fourteen participants (10.8%) had fewer than 20 teeth, while another 13 participants were edentulous in one or both dental arches. Of these, eight individuals had a complete denture in one or both arches, and three others had a partial denture.

**TABLE 1:** Characteristics of the study participants.

	*n* (%)
Total	136 (100)
Indigenous status (self‐identified)	
Aboriginal	124 (91.2)
Torres Strait Islander	0
Aboriginal and Torres Strait Islander	4 (2.9)
Other/undisclosed	8 (5.9)
Gender	
Female	80 (58.8)
Male	56 (41.2)
Age at enrolment (years)	
Median (IQR)	46 (32.0, 55.3)
< 30	30 (22.1)
31–45	38 (27.9)
46–60	45 (33.1)
> 60	23 (16.9)
Location	
Metropolitan and inner regional	117 (86.0)
Outer regional, remote, and very remote	19 (14.0)
Appointment mode	
In‐person appointment	129 (94.9)
Remote appointment (no clinical exam)^a^	7 (5.1)
Dentition status^b^	
Fully dentate (28 teeth)	30 (23.3)
20–27 teeth	72 (55.8)
Less than 20 teeth, dentate in both arches	14 (10.8)
Edentulous maxillary and/or mandibular	13 (10.1)
Denture status	
No denture	118 (91.5)
Partial denture(s) (per person)	3 (2.3)
Maxillary and/or mandibular complete denture	8 (6.2)

^a^ Dental characteristics were based on data collected from clinical examinations. Participants who completed follow‐up remotely did not receive dental examinations and were excluded from dentition summaries.

^b^ Dentition status excludes the wisdom teeth (tooth 18, 28, 38 and 48).

Using reflexive thematic analysis, three themes with seven subthemes were generated, highlighting participant stories around denture‐related experiences, with dentures considered to be physical, social and symbolic aspects of oral health experiences (see Table 2).

**TABLE 2:** Themes and subthemes.

Theme	Subthemes
Decision‐making for dentures: Pain, prior treatment and appearance	Need for dentures determined by oral health status: Pain and function Dental treatment received determines choices around dentures “My mouth was a mess”: Dentures are desired for aesthetic concerns
Unmet needs and expectations of dentures	“I haven't been wearing my falsies”: Ill‐fitting dentures Financial investment for dentures undermined by functional problems
Dentures influence social and emotional wellbeing	“It's a big thing”: Dentures impact personal identity Dentures promote confidence

### 3.1 Theme 1: Decision‐Making for Dentures: Pain, Appearance and Prior Treatment

#### 3.1.1 Subtheme: Need for Dentures Determined by Oral Health Status: Pain and Function

Some participants described being unable to sleep or eat adequately due to pain in their mouth, with one participant noting that having no teeth would be “easier” (P235). They wanted to have all their teeth extracted and replaced with dentures to alleviate these difficulties:

I want them [teeth] all pulled out…Yeah I don't want any of em. Cause it's just I'm in so much pain everyday, can't sleep, can't eat much, and yeah it'd be easier just not to have teeth to be honest. Give me those fake ones you know…I want them all pulled. (P235)

Similarly, another participant described how having all their teeth extracted and receiving dentures supported their mouth's typical function and allowed them to eat food more easily:

I basically couldn't eat with the teeth I had so they referred me to you because you were doing the program and thank God [laughs] I got the teeth out and I got dentures in, so at least I can eat now. (P315)

Experiences of pain with existing dentition and difficulties related to function and wellbeing influenced participants' decision‐making around whether they wanted dentures.

#### 3.1.2 Subtheme: Dental Treatment Received Determines Choices Around Dentures

Participants shared how receiving certain dental treatment (e.g., extractions) from dental practitioners shaped their decision‐making around dentures. In some cases, this decision was decided by and imposed upon patients by their dental practitioners, leaving patients with limited choice around whether they could keep healthy teeth:

I had toothache and the dentist there was one of those rogue dentists who actually removed good teeth in an effort to sell me false teeth. So I was very reluctant to go back to any dentist again, and they did a terrible job of it. I actually had pieces of tooth left in my gum and all sorts of things, experienced pain for months afterwards. (P400)

Another participant shared a similar experience, however, in this case, the treatment received was described by the participant to make it harder for them to receive dentures in the future:

I wasn't happy because there were some bits of the bone that came away so that I wasn't able to have dentures in the near future and that was something that didn't get spoken about before the procedure and [dentist] was trying to take out one of the back teeth…because of the way it was taken out, “Oh now you don't get dentures you're going to look after your teeth really good, you're going to have to figure out another way of getting false teeth”, and that was like the decision was already made for me, I wasn't very happy with that. (P339)

In this subtheme, participants described limited autonomy over their choice to receive (or to not receive) dentures, as determined by the dental treatment they received, with decision‐making around this choice often out of their control.

#### 3.1.3 Subtheme: “My Mouth Was a Mess”: Dentures Are Desired for Aesthetic Concerns

In addition to pain, functional aspects and dental treatment, concerns surrounding personal appearance influenced decisions regarding dentures. One participant shared: “I had nothing wrong with my teeth, I just wanted falsies” (P197). For some, absent teeth strongly influenced decision‐making around dentures:

I had to have dentures I had virtually no teeth left…Basically my mouth was a mess it was on its last legs and so something had to be done straight away because the plan was to continue my 49 years I've been in rock'n'roll…I haven't been able to get on stage a) because of COVID b) because I've put on too much weight during COVID and c) because of my teeth…Hopefully next year I can get back on stage and fire up again. (P315)

Overall, this theme suggests that decision‐making around dentures for Aboriginal and Torres Strait Islander adults is guided by the level of pain they experience within their mouth and how this pain influences functional aspects such as chewing food; the dental treatment they receive—whether this accommodates for dentures, or whether practitioners communicate treatment plans and potential outcomes; and aesthetic concerns.

### 3.2 Theme 2: Unmet Needs and Expectations of Dentures

#### 3.2.1 Subtheme: “I Haven't Been Wearing my Falsies”: Ill‐Fitting Dentures

Many participants had trouble with dentures that were ill‐fitting—either too loose that they would fall out, or too large that they were difficult to remove. One participant expressed disappointment with their dentures sharing that it often hurt their mouth and lips when inserting or removing them:

I'm not happy with my teeth, the false teeth…I've got a small mouth they [dentist] tell me… Sometimes I split the side of my lips trying to get the bottom denture in…I gotta use Polident [adhesive for dentures], and I have trouble getting them out. I don't know whether I put too much Polident on, but it splits my mouth. (P357)

Due to discomfort, many did not wear their dentures. One participant shared how both he and his partner were having difficulties with their dentures and would be following up with their dentists:

I haven't been wearing my falsies ever since I got em…I don't feel comfortable with em…I seen [partner] other day, she looked good with them dentures in but saying they're a bit loose and still giving her a bit of trouble. Like when she goes back [to the dentist], I'll probably go and see mines and do follow up on mine…how I felt when I first wore my dentures, yeah it was sore. (P40)

The issues that participants experienced with their dentures resulted in some not wearing their dentures and others experiencing pain. Some continued to follow‐up on these issues with their dentist, however this required multiple visits and tweaking of the prosthetic device.

#### 3.2.2 Subtheme: Financial Investment for Dentures Undermined by Functional Problems

Participants who wanted dentures and subsequently received them shared their frustration regarding the fact that a significant investment was required and that this was compromised by functional problems with their prosthetic device:

I can't even get two words out without [dentures] dropping into my mouth… I'm just not comfortable with the way they sit and feel and the fact that I can't eat properly with them… I'm still feeling like there's parts at the back are rubbing so much that I cannot literally have them in my mouth without them hurting…if I'm gonna be out of pocket eleven hundred bucks, I want something that fits. (P409)

Another participant described how their dentures required multiple adjustments and still did not fit comfortably. Despite functional problems and the high cost, the participant was grateful to have them:

…for the last six months, I've been getting the dentures made and adjusted and adjusted and adjusted and we're still not quite there…I've seen government provides dentures for free for people over 60 or pensioners…every time I look through that, it was not available for me and so I had to pay over a thousand dollars out of pocket, plus the private insurance just to get the dentures I've got. So the dentures aren't perfect, but at least I've got some. (P315)

This theme overall highlights participants' needs and expectations of dentures in providing functional support with essential tasks such as chewing and eating. These needs and expectations were often unmet due to ill‐fitting dentures causing discomfort and pain and requiring multiple adjustments. For some participants, these issues were further amplified by the significant investment required for dentures for which there was little financial support and many found challenging to fund.

### 3.3 Theme 3: Dentures Influence Social and Emotional Wellbeing

#### 3.3.1 Subtheme: “It's a Big Thing”: Dentures Impact Personal Identity

Dentures were described as having noticeable impacts on social and emotional wellbeing for Aboriginal and Torres Strait Islander adults, particularly in relation to personal identity and as something that some adults would require for the rest of their lives. In many cases, this also encompassed impacts to identity around tooth loss or extraction of all teeth prior to receiving dentures:

…like [partner] had unfortunate event where she's lost all her teeth and she's got dentures and stuff like that so that in a whole impacted me and the family and relationships and the whole lot because she obviously struggled with her identity…a big thing to have all your teeth removed have all that work done to be able to know that you're gonna have that for the rest of your life you know, false teeth all the rest of it. (P403)

Participants described the process of losing or having teeth extracted and subsequently receiving dentures as “a big thing” in relation to identity, and in some cases, a positive event:

…it's been a positive outcome…I knew everything was going to be getting fixed up no matter what. Even all your tooth getting moved out and new dentures getting put back on. It's a big thing. And yeah, that's what I've really experienced through these dentures. (P455)

For many, the impacts of dentures on personal identify also included feelings of confidence and improved self‐esteem, as highlighted in the following subtheme.

#### 3.3.2 Subtheme: Dentures Promote Confidence

Participants with dentures described the impacts they felt in relation to feeling more confident with themselves and in social situations. One participant described how he did not want dentures at first, but that these feelings changed after receiving his dentures which met his needs and expectations after consultation with the dentist:

I feel good about it, I didn't at first having to have false things, I wanted [original teeth] to be intact. But the thing is, I'm happy with the teeth, I mean it's perfect, it's exactly how I explained to [dentist] how I wanted it… So people say, "You have nice teeth". (P96)

Similarly, another participant with partial dentures described her satisfaction with the dentures and being able to retain some of her own teeth:

I like it that I've still got a lot of my own teeth as well…my children don't even know I have a denture… it looks exactly the same as the other teeth…they all blended in. You can't even tell which is which. Yeah, that's how good they are. (P259)

Overall, dentures were found to have significant impacts on personal identity and social and emotional wellbeing. This encompassed positive and negative feelings around tooth loss, extraction and replacement with dentures, impacting relationships and self‐esteem.

## 4 Discussion

This study explored Aboriginal and Torres Strait Islander adults' perceptions of and experiences with dentures regardless of denture status, centring their lived experience perspectives and highlighting factors that shape oral rehabilitation. This study highlighted that for participants, decision‐making around extractions and subsequent dentures was determined by how they perceived their oral health status, particularly if they were experiencing impacts to their quality of life. Echoing previous research, participants who experienced severe pain, often as a result of dental caries or periodontal disease, saw extractions and tooth loss as a primary method of pain relief from their ongoing dental problems [5, 27]. Extractions or tooth loss were also seen as means to receiving further oral rehabilitation in the form of dentures to support oral health and quality of life.

When accessing general dental care such as routine check‐ups, some Aboriginal and Torres Strait Islander participants in this study highlighted a lack of patient autonomy and medical paternalism. Obtaining informed consent to procedures or treatment is a fundamental aspect of healthcare—one which some participants felt was not ensured or acknowledged by practitioners. Often, this meant that clinician decisions overrode patient preferences, for example where teeth were prematurely removed by practitioners and appropriate education or alternatives were not discussed with participants, as reflected in prior international literature [5, 28].

Decision‐making around dentures for Aboriginal and Torres Strait Islander adults was also driven by their concerns around their appearance after losing or having teeth removed. Similar to participants in a previous Australian study who received complete removable dentures, dentures were seen by Aboriginal and Torres Strait Islander adults in the current study as therapeutic devices alleviating both pain and aesthetic concerns [29]. Overall, participants in the current study shared that their choices around receiving dentures were influenced by pain, prior treatment and appearance.

Another key finding of this study were perceptions around the unmet needs and expectations of the denture experience. Mirroring previous qualitative research, some participants in this study reported negative experiences in the adjustment period including ongoing oral discomfort with dentures that were unstable, easily dislodged or not the right size, which influenced how frequently or whether they wore the device [27, 28, 30]. Despite these unmet needs, many participants wanted to achieve denture retention and stability, reflecting their resilience in adapting to these limitations and in coping with the multifaceted challenges that come with impacted oral function [28].

Another key finding of our study reported by participants was the significant financial investment required to receive dentures, further amplified by functional issues such as ill‐fitting prosthesis and systems‐level factors. In South Australia, costs vary depending on the type of denture required, the materials used, whether patients are seen by dentists or prosthetists and whether they are eligible for subsidised care [20]. The average cost of complete dentures in Australia ranges from $2000 to $5000 AUD, with partial dentures roughly costing $750 to $2800 AUD [31]. These fees may be partially subsidised depending on whether patients have private health insurance or government healthcare cards such as the Pensioner Concession Card. They also do not account for other costs involved such as extractions, consultations, or follow‐ups [31]. In a cost analysis conducted as part of our overarching study, many participants reported that paying a $100 AUD dental bill (significantly below the average expenditure for prosthetic care) would be a bit hard, very hard, or not possible [32]. These experiences highlight issues related to the affordability of dental care more broadly, which limit access to essential, preventive care for Aboriginal and Torres Strait Islander Australians.

A final key finding of this study was the noticeable impacts tooth loss and subsequent dentures had on wellbeing for Aboriginal and Torres Strait Islander adults, specifically in relation to their personal identity and self‐confidence. For many Aboriginal and Torres Strait Islander communities, wellbeing is viewed holistically and is conceptualised as social and emotional wellbeing with domains centring healthy connections to body, mind and emotions, family and kinship, community, culture, Country and spirituality [33]. Participants reported that tooth loss had significant personal and interpersonal impacts extending to the mind and emotions and family and kinship domains of social and emotional wellbeing. As such, the process of tooth loss and subsequent oral rehabilitation typically entailed a level of personal trauma that had lasting emotional impacts, reflecting prior research [10]. Further highlighting the multifaceted social and emotional experience of living with dentures, many participants in this study noted improved self‐confidence and self‐image particularly in social settings—commonly reported in the broader literature as positive implications of denture treatment [34, 35].

### 4.1 Clinical Implications

Experiences from this study highlight clinical implications that may better support oral healthcare for Aboriginal and Torres Strait Islander adults, particularly in relation to oral rehabilitation. Firstly, a key experience participants oriented to was a lack of patient autonomy around dental decision‐making, illustrating a need for patient‐centred care that prioritises cultural safety including hearing and understanding patient concerns, and clear explanation of processes by practitioners [18]. Participants also emphasised the significant financial investment required for dentures—for which they had hoped would improve factors such as aesthetics and function—many of whom found it challenging to fund. The dominance of privately provided dental care in Australia may be a factor in ongoing socioeconomic inequities in relation to tooth loss and denture wearing [1]. Additionally, limited prioritisation of oral rehabilitation and denture treatment within government schemes as well as considering dentures a ‘cosmetic luxury’ may be further barriers. These experiences emphasise the need for oral healthcare services that centre and prioritise: local needs; cultural safety at all levels of staffing, environment and service provision; clear approaches to promoting and sustaining tailored services including those that focus on oral rehabilitation; and Indigenous governance (e.g., cultural authority, local knowledge) [36].

### 4.2 Limitations and Future Research

While this study was one of the first to consider denture‐related experiences among Aboriginal and Torres Strait Islander Australian adults, we note some limitations and recommendations for future research. Firstly, this study did not disaggregate participants' experiences based on their denture status (i.e., partial, complete, implants). This is an important avenue for future research to explore given the potential variation in treatment experiences. The study did not specifically focus on Aboriginal and Torres Strait Islander communities living in regional and remote areas who may experience additional barriers in accessing oral healthcare (e.g., delayed care and underservicing), and who may therefore have varied experiences regarding denture decision‐making and care. Future qualitative studies should explore this further, as well as oral health related quality of life and oral health impacts regarding other aspects of life (e.g., sleep, employment, eating).

## 5 Conclusion

The denture‐related experiences described by Aboriginal and Torres Strait Islander adult participants in this study were interrelated, from prosthetic decision‐making to the unmet needs and expectations of dentures, and finally, the impact of tooth loss and subsequent oral rehabilitation on social and emotional wellbeing. The strength and capacity of participants in overcoming challenges related to tooth loss and denture‐wearing such as pain and impacts to identity were salient. Nonetheless, consideration of equitable approaches to oral rehabilitation are required, including making prosthetic care more accessible and affordable, prioritising patient‐centred care in supporting autonomy and centring quality of life to promote social and emotional wellbeing.

## Funding

This research was funded by the National Health and Medical Research Council (grant no. APP1120215). Lisa M. Jamieson is supported by the NHMRC Research Fellowship (grant no. APP1102587). The funding body did not have any role in designing the study, collecting, analysing or interpreting the data or in writing the manuscript.

## Conflicts of Interest

The authors declare no conflicts of interest.

## Supporting information


**Data S1:** CONSIDER statement.

## Data Availability

Research data are not shared.
