# Supplementary material for: ‘I Got Dentures in, So at Least I Can Eat Now’: Aboriginal and Torres Strait Islander Peoples' Perceptions of and Experiences With Removable Dentures
Source: Health Promot J Austr. 2026 Oct 20;38(1):e70259. doi: 10.1002/hpja.70259 (PMC13682950; doi:10.1002/hpja.70259)
Supplement: Supplementary file 1 [file HPJA-38-0-s001.docx]

| **Governance** | |
| --- | --- |
| 1. | *Describe partnership agreements between the research institution and Indigenous-governing organization for the research, (e.g., Informal agreements through to MOU (Memorandum of Understanding) or MOA (Memorandum of Agreement)).*   - Formal letters of support provided by Sovereign Aboriginal organisations that the chief investigator and Senior Aboriginal Research Officer had connections with, including Chief Executive Officers and leaders within key South Australian Aboriginal Community Controlled Health Organisations (ACCHOs). |
| 2. | *Describe accountability and review mechanisms within the partnership agreement that addresses harm minimization.*   - Ethics approval granted by the Aboriginal Health Council of South Australia Human Research Ethics Committee and the University of Adelaide Human Research Ethics Committee. - Letters of support provided by CEOs of South Australian ACCHOs in relation to the research procedures, and protocols. - Study oversight was provided by an Indigenous Oral Health Unit Reference Group (IOHURG), which offered guidance across all project dimensions including staff recruitment, participant engagement, data collection protocols, analytical processes, and community feedback mechanisms. |
| 3. | *Specify how the research partnership agreement includes protection of Indigenous intellectual property and knowledge arising from the research, including financial and intellectual benefits generated (e.g., development of traditional medicines for commercial purposes or supporting the Indigenous community to develop commercialization proposals generated from the research).*   - Indigenous governance rights are protected by the partnership between the Indigenous Oral Health Unit and key ACCHOs. - Informed consent was obtained from all study participants prior to their involvement in the research. - The project received ongoing guidance from the IOHURG, the Aboriginal Research Officers, and ACCHO connections. |
| **Prioritization** | |
| 4. | *Explain how the research aims emerged from priorities identified by either Indigenous stakeholders, governing bodies, funders, non-government organization(s), stakeholders, consumers, and empirical evidence.*   - An established body of evidence highlights poorer overall wellbeing and oral health among Indigenous Australians compared to non-Indigenous Australians. - This includes a lack of culturally safe oral healthcare and several barriers to accessing dental services, as identified in the broader literature and by Indigenous leaders within South Australian ACCHOs. - The intervention aims included promoting: quality of life; self-confidence through supporting communities with dental aesthetics and function; productivity in school or workplaces; and better prognosis for chronic diseases linked with poor oral health. Qualitative understandings can shape health services planning and delivery of culturally safe dental care. |
|  | **Relationships** (Indigenous stakeholders/participants/research team) |
| 5. | *Specify measures that adhere and honor Indigenous ethical guidelines, processes, and approvals for all relevant Indigenous stakeholders, recognizing that multiple Indigenous partners may be involved, e.g., Indigenous ethics committee approval, regional/national ethics approval processes*.   - Ethics approval granted by the Aboriginal Health Council of South Australia Human Research Ethics Committee and the University of Adelaide Human Research Ethics Committee. - Research aims and protocols reviewed and endorsed by the ACCHOs and Aboriginal health services involved, and the IOHURG. |
| 6. | *Report how Indigenous stakeholders were involved in the research processes (i.e., research design, funding, implementation, analysis, dissemination/recruitment).*   - Research design and implementation informed by a Senior Aboriginal Research Officer, Indigenous academics and the IOHURG. - Ongoing and longstanding relationship between the Indigenous Oral Health Unit, ACCHOs and Aboriginal health services who supported with dissemination and recruitment. - Study oversight was provided by the IOHURG, which offered guidance across all project dimensions including staff recruitment, participant engagement, data collection protocols, analytical processes, and community feedback mechanisms. |
| 7. | *Describe the expertise of the research team in Indigenous health and research*   - The Indigenous and non-Indigenous research team have over 20 years combined experience working with Indigenous communities in the oral health field. - Dr Nicolas Reid is a proud member of the Dharug nation and general dentist with extensive experience working with Indigenous communities in Australia. This work has included travelling to remote communities in South Australia and the Northern Territory to provide essential dental services, as well as working with ACCHOs. Dr Reid is also involved in a longitudinal study exploring oral HPV and oropharyngeal cancer in Indigenous Australian adults and oral microbiome research. - Chief Investigator Prof Lisa Jamieson is a dentist with a strong focus on Indigenous oral health and Indigenous research engagement, embedded in a world-class research centre (Indigenous Oral Health Unit), with strong connections to Aboriginal health service providers and policy makers. - Along with oral health therapist Dr Kostas Kapellas, Prof Jamieson and Dr Kapellas have led and conducted various interventions involving comprehensive dental care for Indigenous Australians, including collection of biodata to assess clinical biomarkers before and after dental intervention. - Dr Gina L. Guzzo is a microbiome scientist with extensive experience in the coordination of fieldwork, dental charting, microbial sample collection, point-of-care testing, and oral health communication with Indigenous study participants and community members in metropolitan and regional South Australia. - Ms Johanna Groundwater is an oral health therapist with experience in Indigenous health research and in providing oral healthcare for Indigenous communities in regional, remote, and very remote Australia. - Dr Sonia Nath is a dentist with over 5 years of experience engaging with Indigenous communities through culturally safe oral health research practices. This includes leading the current project providing culturally safe dental care for over 400 Indigenous Australian participants, overseeing and conducting clinical examinations for Indigenous communities in metropolitan and regional South Australia. - Dr Ria Aiyar has sound experience in conducting qualitative research in relation to improving health systems and promoting wellbeing. Dr Aiyar has been involved in leading the 60-month follow-up phase of the longitudinal study exploring oral HPV and oropharyngeal cancer in Indigenous South Australian adults. |
|  | **Methodology** |
| 8. | *Describe the methodological approach of the research including a rationale of methods used and implication for Indigenous stakeholders, e.g., privacy and confidentiality (individual and collective)*   - Thematic analysis was used to analyse the qualitative data, as this approach allowed us to center the lived experiences of participants in terms of their wellbeing and oral health experiences. - All qualitative data was de-identified to ensure privacy and confidentiality. - The Aboriginal Research Officers and Aboriginal dental professional and researcher (NR) provided ongoing guidance throughout data generation and analysis. |
| 9. | *Describe how the research methodology incorporated consideration of the physical, social, economic and cultural environment of the participants and prospective participants. (e.g., impacts of colonization, racism, and social justice). As well as Indigenous worldviews.*   - The research centered health equity in considering the impacts of colonisation, racism and social justice on participants’ oral health and healthcare experiences. - Considering the social and emotional wellbeing model within the current research praxis, health is viewed as holistic, the importance of kinship is recognised, and Aboriginal and Torres Strait Islander strengths are centered. |
|  | **Participation** |
| 10. | *Specify how individual and collective consent was sought to conduct future analysis on collected samples and data (e.g., additional secondary analyses; third-parties accessing samples (genetic, tissue, blood) for further analyses).*   - Data that is published is summary data and is de-identified. - Data is securely stored and kept according to ethics protocols, with all access logged requiring multi-factor authentication and limited to research staff. - Participants involved in the research own the data. |
| 11. | *Described how the resource demands (current and future) placed on Indigenous participants and communities involved in the research were identified and agreed upon including any resourcing for participation, knowledge, and expertise.*   - In limiting resource demands, participants could partake in oral epidemiological examinations at a date, time, and location of their choosing (e.g., their home, at ACCHOs, a University research facility). - Participation in qualitative interviews was voluntary and participants could withdraw at any time during the follow-up. The option to complete the interview over the phone at a later time was also offered. |
| 12 | *Specify how biological tissue and other samples including data were stored, explaining the processes of removal from traditional lands, if done, and of disposal.*   - Qualitative data is securely stored and kept according to ethics protocols with all access logged requiring multi-factor authentication and limited to research staff. |
|  | **Capacity** |
| 13. | *Explain how the research supported the development and maintenance of Indigenous research capacity (e.g., specific funding of Indigenous researchers).*   - Aboriginal Research Officers and the Aboriginal dental professional provided ongoing guidance to non-Indigenous members of the research team regarding culturally safe practices. |
| 14. | *Discuss how the research team undertook professional development opportunities to develop the capacity to partner with Indigenous stakeholders?*   - All researchers engaged in education regarding culturally safe research practices, as well as ongoing discussions around yarning about oral health and wellbeing with Indigenous communities. These development opportunities were led by Aboriginal Research Officers within the IOHU. - Dr Reid has extensive experience providing guidance to both Indigenous and non-Indigenous researchers regarding safe research practices, as well as information around Indigenous oral health and wellbeing. |
|  | **Analysis** **and** **interpretation** |
| 15. | *Specify how the research analysis and reporting supported critical inquiry and a strength-based approach that was inclusive of Indigenous values.*   - Analysis reflected the positive outcomes the intervention had in terms of addressing aims around promoting functional aspects of oral health, supporting chronic disease management, and addressing inequities in health and healthcare. - Additionally, the social and emotional wellbeing model were considered within the current research praxis, whereby Aboriginal and Torres Strait Islander strengths were centered, and health viewed holistically. |
|  | **Dissemination** |
| 16. | *Describe the dissemination of the research findings to relevant Indigenous governing bodies and peoples.*   - The study team disseminated research update newsletters to all participants, and Indigenous stakeholder groups including the involved ACCHOs. Results will also be published in academic journals. - Findings will also be shared with key stakeholders at Indigenous health and oral health conferences held nationally and internationally. |
| 17. | *Discuss the process for knowledge translation and implementation to support Indigenous advancement (e.g., research capacity, policy, investment).*   - The project has various important outcomes. This particular study provides empirical evidence of how best to support and promote oral health across the lifespan for Indigenous communities. More broadly, this is important for health services planning in the dental (private and public sector) as well as the ACCHO sector, where the management of dental diseases to support wellbeing and improve chronic disease outcomes is currently insufficiently understood, planned and budgeted for. |
